# Turkish Adaptation of the Feeding Practices and Structure Questionnaire Milk Feeding and Solid Feeding Versions

**DOI:** 10.1002/fsn3.70357

**Published:** 2025-05-27

**Authors:** Mahmut Caner Us, Hatice Ezgi Baris, Elif Öztürk, Ayşe Şahin, Gökçe Nizam, Betül Şenyürek, Perran Boran

**Affiliations:** ^1^ Division of Social Pediatrics, Department of Pediatrics, School of Medicine Marmara University Istanbul Turkey; ^2^ Institute of Health Sciences Marmara University Istanbul Turkey

**Keywords:** adaptation, feeding practices, infants, questionnaire, responsive feeding, structured mealtimes, toddlers

## Abstract

This study aimed to conduct a Turkish language adaptation of the Feeding Practices and Structure Questionnaire Milk Feeding (FPSQ‐M) and (Semi‐)Solid Feeding versions (FPSQ‐S), which were developed to assess dietary content and parental feeding practices in early childhood, for use in our country. Subjects were recruited from an internet sample through social media. Internal consistency was assessed by Cronbach's alpha and test–retest reliability by Pearson's correlation test and paired *t*‐test. Principal component factor analysis was used for factorial validity. A total of 411 parents with children aged 0–24 months participated in the study (*n*: 181 for the FPSQ‐M, *n*: 224 for the FPSQ‐S). The Cronbach's alpha coefficient for the FPSQ‐M was found to be 0.78 and the four‐component model accounted for 61.69% of the total variance, with all four subscales showing moderate to good internal consistency (using food to calm: 0.88, parent‐led feeding: 0.81, persuasive feeding: 0.79, and feeding on demand: 0.66). The Cronbach's alpha coefficient for the FPSQ‐S was found to be 0.87, and the six‐component model accounted for 59.71% of the total variance, with all six subscales showing moderate to good internal consistency [using (non‐)food rewards: 0.89, using food to calm: 0.85, persuasive feeding: 0.83, family meal environment: 0.83, feeding on demand: 0.68, and parent‐led feeding: 0.60]. Both versions demonstrated significant and moderate to strong test–retest reliability. The Turkish adapted FPSQ‐M with 16 items distributing a four‐factor structure and the FPSQ‐S with 31 items distributing a six‐factor structure were reliable questionnaires for measuring feeding practices among Turkish children aged 0–24 months.

## Graphical abstract

Culturally adapted feeding questionnaires help healthcare providers guide parents toward responsive feeding practices and encourage recognition of hunger and satiety cues to promote healthy eating habits. The Turkish‐Adapted Feeding Practices and Structure Questionnaire‐Milk Feeding version with a 16‐item distribution, a four‐factor structure, and the (Semi‐)Solid Feeding versions with a 31‐item distribution, a six‐factor structure, are reliable questionnaires for measuring feeding practices among Turkish children aged 0–24 months. These questionnaires provide valuable insights into culturally specific feeding practices.

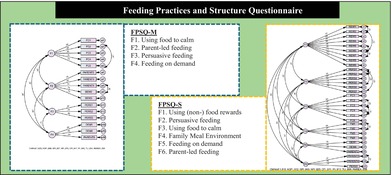

## 1 Introduction

The first 1000 days of life represent a critical period for child development. Dietary practices and habits during this period influence adult dietary behaviors (Mikkila et al. 2005; Woo Baidal et al. 2016). Recent population surveys in Türkiye have shown that 8.1% of children under 5 years of age are overweight, 2% are wasted, and 6% are stunted (Hacettepe Üniversitesi Nüfus Etütleri Enstitüsü 2021). Given the short‐and long‐term health consequences, feeding practices during this early period need to be comprehensively addressed to ensure optimal nutritional outcomes.

Promoting early childhood development requires following the principles of nurturing care, which includes responsive caregiving, adequate nutrition, and good health practices (World Health Organization 2018). Despite the 2030 global targets to improve early childhood development, mixed progress has been observed. Although the reductions in stunting and wasting remained lower than expected, levels of overweight have persisted or increased in some regions, raising significant concerns (World Health Organization et al. 2021).

Improving parental feeding practices like parental responsiveness to infant hunger cues has been shown to be the most promising behavioral intervention for reducing overweight and obesity under 2 years of age (Redsell et al. 2016). Furthermore, positive parenting practices have been linked to healthy food preferences in children. Children with authoritative parents tend to consume more fruits compared to children with authoritarian or permissive parents (Chen et al. 2021).

In addition to content, timing, and quantity, complementary feeding guidelines emphasize the behavioral and relational dimension of feeding. These include preferring non‐food‐based strategies over rewards and punishments and focusing on the behavioral and relational aspects of feeding, such as caregiver‐child interaction and responsive care (Kerzner et al. 2015; World Health Organization 2023). Apart from behavioral strategies suggested for parents, other studies focused more on the family environment, highlighting the crucial role of parents as positive role models. For instance, Möhler et al. suggested interventions which promote parents' self‐efficacy, outcome expectations and parental healthy nutrition to strengthen children's healthy eating habits (Mohler et al. 2020).

Particularly during the transition to complementary feeding, appropriate feeding interaction between mother and infant can influence infant feeding behavior. However, in addition to the infant's temperament, the mother's cultural beliefs about feeding may also be important in the development of feeding problems (Kerzner et al. 2015; Santona et al. 2015). In Türkiye, parenting is often characterized by high emotional warmth alongside strong parental control aimed at ensuring obedience, a pattern that may extend to feeding interactions (Kagitcibasi 2010; Sen et al. 2013). This dynamic may contribute to more controlling feeding practices, such as pressuring children to eat, which may hinder the development of self‐regulation and increase the risk of childhood obesity, as also observed in Turkish toddlers and preschoolers exposed to authoritarian parenting styles (Melis Yavuz and Selcuk 2018; Sarahman‐Kahraman et al. 2024). However, when parents adopt more authoritative and responsive approaches, healthier eating behaviors tend to emerge. Furthermore, evidence suggests that supporting mothers, actively involving fathers in caregiving, and providing parents with access to reliable, evidence‐based guidance during the complementary feeding period can ease the transition and promote more positive long‐term eating habits (Demirel Ozbek et al. 2025).

A detailed assessment of feeding problems is important to provide families with appropriate interventions. The assessment should include a detailed physical examination, including anthropometric measurements, medical history, feeding diary, and an assessment of the caregiver approach with validated measures (Chatoor et al. 2022). There is a need for culturally validated tools to understand deeper families' beliefs about child feeding, cue responsiveness, parental feeding practices, and children's eating habits. It has been shown that as early as the interventions delivered result in more positive caregiver‐child feeding interactions (Globus et al. 2019). Studies and measures that objectively assess the feeding practices of caregivers are limited (Chatoor et al. 2022; Squires et al. 2014). Inconsistent and incomplete measurement tools have limited our understanding of the developmental role and impact of parental feeding practices. To this end, the Feeding Practices and Structure Questionnaire (FPSQ) for Infants (Milk Feeding version) and Toddlers (Semi‐Solid Feeding version) was developed by Jansen et al. (2021) to assess parental feeding content and practices. The FPSQ provides a prospective understanding of how these behaviors influence children's eating habits and weight outcomes during early childhood. It also makes it possible to examine how parental feeding practices evolve over time across different parent–child dyads and feeding modes (breastfeeding, bottle feeding, solid foods, and family foods), and how these practices affect eating behavior and weight outcomes. Examining parental practices is particularly important in Türkiye, since most tools are developed in Western cultural context. Cultural validation is essential to gain further insight into Turkish parents' feeding practices. Compared to other instruments, both versions of the FPSQ provide health professionals and researchers with a unique opportunity to measure parental feeding practices across infancy and childhood. In addition, both versions can be used to identify underlying processes, thereby providing guidance to parents/society on how to promote healthy eating to prevent nutritional problems such as obesity later in life.

The primary aim of this study was to the language adaptation of the FPSQ into Turkish. The secondary aim was to assess the construct validity of the FPSQ through convergent validity analysis, by administering the “IOWA Infant Feeding Attitude Scale (IIFAS)” (Eksioğlu et al. 2016; Mora et al. 1999) and the “Parental Feeding Style Questionnaire (PFSQ)” (Özçetin et al. 2010; Wardle et al. 2002).

## 2 Methods

### 2.1 Subjects and Procedure

This study was conducted between March and September 2024, targeting parents of infants aged 0–24 months. Participants were recruited via online platforms such as social media groups as well as in‐person at the Well‐Child Outpatient Clinic.

Parents over 18 years of age with infants aged 0–24 months were invited to participate. Inclusion criteria required parents to be literate and have an infant born ≥ 37 weeks of gestation, weighing over 2500 g at birth, without a diagnosed feeding disorder. Parents of infants with chronic health conditions or those who did not provide consent were excluded.

The sample sizes for both versions of the questionnaires surpassed the minimum recommended respondent‐to‐item ratio of 5:1–10:1 for factor analyses, ensuring stable and reliable psychometric evaluations (Comrey and Lee 2013; Tabachnick et al. 2013). To test the temporal stability of the questionnaires, 30 participants who provided consent and shared their contact information completed the questionnaires again 3 weeks after the initial survey.

### 2.2 Tools

Data were collected using self‐administered online questionnaires hosted on Google Forms. The questionnaire comprised two main sections. The first section was an informed consent form; participants who marked all checkboxes as “I agree” were granted access to the second section. Participants who did not provide consent were directed to a thank you page, and their access to the questionnaire was restricted. A digital copy of the consent form was emailed to participants upon request.

Separate Google Forms were created for the two subscales of the FPSQ: the FPSQ‐M for infants aged 1–6 months and the FPSQ‐S for children aged 6–24 months. The initial instructions clarified the target age group for each questionnaire, allowing participants to proceed to the consent form only after confirming their infant's age. The questionnaire for infants aged 0–6 months included a sociodemographic data form, FPSQ‐M, and the IIFAS. For children aged 6–24 months, the questionnaire included a sociodemographic data form, FPSQ‐S, and the PFSQ. All questions in the questionnaires were set as mandatory, which did not allow participants to proceed to the next section or to send the Google Form to avoid missing data.

#### 2.2.1 Sociodemographic Data Questionnaire

This section collected the prenatal, natal, postnatal, personal, and family histories of the participants.

#### 2.2.2 Feeding Practices and Structure Questionnaire

The FPSQ, developed by Jansen et al. (2021), has two versions tailored to different age groups: FPSQ‐M, for infants aged 0–6 months, with 18 items, and FPSQ‐S, for children aged 6–24 months, with 34 items. The 18 items in the milk‐feeding version of the original questionnaire were grouped into four factors. The subfactors are: feeding on demand (*α* = 0.87), parent‐led feeding (*α* = 0.79), persuasive feeding (*α* = 0.71), and using food to calm (*α* = 0.87). The (Semi‐)Solid Feeding version consists of 21 items and the same four factors. Cronbach's alpha values were 0.74, 0.86, 0.85, and 0.84, respectively. Two additional factors (13 items) were developed for the (semi‐)solid food version, which appears to be developmentally appropriate only for children aged 12 months and older. The additional factors were the family meal environment (*α* = 0.81) and the using (non‐)food rewards (*α* = 0.92). All items were scored on a 5‐point Likert scale ranging from 1 = never to 5 = always. The response option “not applicable” is available for all items. Items marked with * are reverse scored. An average score is calculated for each sub‐factor [e.g., feeding on demand average score = (DEM1 + DEM2 + DEM3 + DEM4)/4]. For the milk feeding version, the maximum score is 20 and the minimum score is 4. For the (Semi‐)Solid Feeding version, the maximum score is 30 and the minimum score is 6. Lower scores indicate more problematic feeding behavior (Jansen et al. 2021). The Turkish adaptation of FPSQ was performed using independent forward and backward translations, and cultural adaptation approval was obtained from the original developer.

#### 2.2.3 IOWA Infant Feeding Attitude Scale

The IIFAS, developed by De La Mora and Russell, is a validated tool for assessing maternal attitudes toward breastfeeding and feeding practices, identifying those at risk of early cessation, and evaluating partner or relative attitudes (Mora et al. 1999). Reliability in the original study was Cronbach alpha = 0.86. All 17 items are rated on a 5‐point Likert scale (1 = Strongly Disagree to 5 = Strongly Agree). Scores range from 17 to 85, categorizing attitudes toward breastfeeding or formula feeding. Total IIFAS scores can be further categorized into the following groups: (1) a positive attitude toward breastfeeding (IIFAS score of 70–85), (2) a neutral attitude (IIFAS score of 49–69), and (3) a positive attitude toward formula feeding (IIFAS score of 17–48). The Turkish version was validated by Kezdoglou A. (Cronbach alpha = 0.71) (Özçetin et al. 2010).

#### 2.2.4 Parental Feeding Style Questionnaire

The PFSQ was developed by J. Wardle and consists of 27 items across four subscales: Emotional feeding (*α* = 0.83), Instrumental feeding (*α* = 0.67), Prompting/encouragement (*α* = 0.74), and Control over eating (*α* = 0.81) (Wardle et al. 2002). All items are scored on a 5‐point Likert scale (from 1 = I never do to 5 = I always do). An average score is calculated for each sub‐factor, with a minimum score of 4 and a maximum score of 20. The Turkish version, validated by Ossetin M., identified five subscales after adaptation (Özçetin et al. 2010).

### 2.3 Analysis

Statistical analyses were performed using SPSS Version 28.0 and AMOS (IBM Inc., United States). The sample sizes for both versions exceed the recommended minimum respondent‐to‐item ratio of 5:1–10:1 for factor analyses, supporting the stability and reliability of the psychometric evaluations (Comrey and Lee 2013; Tabachnick et al. 2013). Continuous sociodemographic data are expressed as mean ± standard deviation or median (interquartile range), depending on whether they are normally distributed or not, and categorical data are expressed as percentages (*n* number). For reliability analyses, internal consistency was evaluated using Cronbach's alpha, and test–retest reliability was assessed through Pearson's correlation and paired *t*‐test. For factorial validity, principal component factor analysis was conducted for the components of both the Milk Feeding and (Semi‐)Solids Feeding versions of the FPSQ. Subscales were constructed based on factor loadings, with a difference of > 0.10 considered significant for loadings on different factors. Confirmatory factor analysis (CFA) was performed to assess the distribution of questions across factors. Items with factor loadings below 0.30 were excluded. The model fit was evaluated using the chi‐squared value normalized by degrees of freedom (CMIN/df), the root mean square error of approximation (RMSEA), the comparative fit index (CFI), the incremental fit index (IFI), the Tucker–Lewis index (TLI), the adjusted goodness‐of‐fit index (AGFI), and the goodness‐of‐fit index (GFI) (Byrne 2013; Hu and Bentler 1999).

## 3 Results

### 3.1 Sociodemographic Variables of Participants

A total of 411 parents aged 31.61 ± 4.44 years for the FPSQ‐M and 32.7 ± 5.1 years for the FPSQ‐S (89.3% for the FPSQ‐M and 90.2% for the FPSQ‐S were mothers) participated in the study. Characteristics of the participating parents and their infants are presented in Table 1. Infants' age ranged between 0 and 24 months, with a median of 3 months for FPSQ‐M and 13 months for FPSQ‐S version.

**TABLE 1:** Sociodemographic variables of participants.

	FPSQ‐M (*n*: 187)	FPSQ‐S (*n*: 224)
Age (month), median (min–max)	3 (0–6)	13 (6–24)
Gender, *n* (%)		
Girl	98 (52.4)	124 (50.8)
Boy	89 (47.6)	120 (49.2)
Interviewer, *n* (%)		
Mother	167 (89.3)	220 (90.2)
Father	20 (10.7)	24 (9.8)
Age of parent (year), mean ± SD	31.61 ± 4.44	32.7 ± 5.10
Parental year of education, mean ± SD	15.36 ± 3.99	15.8 ± 3.60
Parental occupation, *n* (%)		
Professional	82 (43.9)	115 (47.1)
Non‐Manuel skilled	37 (19.8)	48 (19.7)
Manuel skilled	3 (1.6)	1 (0.4)
Partly skilled	4 (2.1)	5 (2)
Non‐skilled	4 (2.1)	4 (1.6)
Housewife	46 (24.6)	50 (20.5)
Other	11 (5.9)	21 (8.6)
Employment status of parent, *n* (%)		
Working	63 (33.7)	113 (46,3)
Not working	61 (32.6)	92 (37,7)
On leave	63 (33.7)	39 (16)
Marital status, *n* (%)		
Married	187 (100)	240 (98.4)
Number of children, median (min–max)	1 (0–6)	1 (0–4)
Family size, median (min–max)	3 (2–7)	3 (1–6)

### 3.2 Reliability and Factor Analysis

#### 3.2.1 Reliability and Factor Analysis of FPSQ‐M Version

The psychometric evaluation of the Turkish version of the FPSQ‐M revealed strong results. The Kaiser–Meyer–Olkin (KMO) measure of sampling adequacy was calculated as 0.781, indicating a moderately good level of adequacy for factor analysis (Kaiser 1974). Additionally, Bartlett's test of sphericity demonstrated statistically significant correlations among variables (*p* < 0.01), confirming the appropriateness of the dataset for factor analysis (Bartlett 1950). The internal consistency of the FPSQ‐M, assessed using Cronbach's alpha, was 0.78, indicating acceptable reliability (Cronbach 1951; Mallery and George 2000).

The four‐component model of FPSQ‐M accounted for 61.42% of the total variance. Individual components contributed 23.71% (23.71%), 17.96% (41.67%), 13.00% (54.67%), and 7.03% (61.69%) of the variance, respectively. The four components identified demonstrated varying levels of internal consistency as measured by Cronbach's alpha: for using food to calm = 0.88 (indicating excellent reliability), for parent‐led feeding = 0.81 (indicating good reliability), for persuasive feeding = 0.79 (indicating acceptable reliability), and for feeding on demand = 0.66 (indicating moderate reliability) (Tables 2a and 2b).

**TABLE 2a:** Rotated component matrix FPSQ‐M.

	C‐1	C‐2	C‐3	C‐4	KMO	Com	Cronbach's alpha	Cronbach's alpha after CFA^b^
FPSQ‐M Bartlett's test of sphericity					0.781 < 0.01	—	0.79	0.78
FC 3. I offer my baby a feed when he is hurt	**0.855**.				0.805	0.774	0.88	0.88
FC 2. I offer my baby a feed when he is unsettled or crying	**0.836**				0.805	0.756		
FC4. When my baby gets unsettled or is crying, feeding him is one of the first things I do	**0.816**				0.803	0.684		
FC 5. I feed my baby to make sure that he does not get unsettled or cry	**0.753**				0.809	0.610		
FC 1. I feed my baby to settle him, even if he is not hungry	**0.749**				0.884	0.589		
PARENT 2. I feed my baby for a set time		**0.832**			0.772	0.709	0.81	0.81
DEM 3. I decide when it is time for my baby to have a feed^a^		**−0.763**			0.816	0.595		
DEM 2. I feed my baby at set times^a^		**−0.747**			0.771	0.582		
PARENT 6. I decide how much my baby feeds		**0.744**			0.808	0.635		
PARENT 4. I follow a rule about how much my baby should feed		**0.619**			0.812	0.481		
PARENT 3. I carefully control how much my baby feeds^b^		**0.524**		0.466	0.744	0.492		
PERS 3. I feed my baby extra milk so he sleeps longer			**0.862**		0.652	0.772	0.79	0.79
PERS 2. If my baby indicates he is not hungry, I try to get him to feed anyway			**0.797**		0.748	0.675		
PERS 1. I feed my baby extra milk, just to make sure he gets enough			**0.779**		0.781	0.649		
DEM 4. I let my baby decide when he would like to have a feed				**0.797**	0.687	0.662	0.66	0.65
PARENT 5. I let my baby decide how much he feeds^a^				**−0.692**	0.687	0.520		
DEM 1. I feed my baby whenever he wants	0.325			**0.605**	0.772	0.482		
PARENT 1. When deciding how much to feed my baby, I rely on how hungry he is^a^, ^b^		−0.371		**−0.527**	0.761	0.438		

*Note:* Bold values indicate the subgroup to which they belong.

Abbreviations: C, component; CFA, confirmatory factor analysis; Com, communalities; DEM, feeding on demand; Extraction Method, Principal Component Analysis; FC, using food to calm; FPSQ‐M, Feeding Practices and Structure Questionnaire milking version; KMO, Kaiser–Meyer–Olkin measure of sampling adequacy; PARENT, parent‐led feeding; PERS, persuasive feeding; Rotation Method, Varimax with Kaiser normalization.

^a^ Item is reverse coded.

^b^ Parents 1 and 3 was excluded after CFA.

#### 3.2.2 Reliability and Factor Analysis of FPSQ‐S Version

The psychometric evaluation of the Turkish version of the FPSQ‐S also revealed strong results. The internal consistency of the FPSQ‐S, as determined by Cronbach's alpha coefficient, was 0.87, indicating very good reliability (Cronbach 1951; Mallery and George 2000). The six‐component model of FPSQ‐S accounted for 59.52% of the total variance, with individual components contributing 24.44% (24.44%), 10.69% (35.14%), 8.83% (43.97%), 6.12% (50.10%), 5.17% (55.27%), and 4.27% (59.71%), respectively. For the FPSQ‐S version, six components were identified with the following Cronbach's alpha values: feeding on demand = 0.68, using food to calm = 0.85, persuasive feeding = 0.83, parent‐led feeding = 0.60, family meal environment = 0.83, and using (non‐)food reward = 0.89 (Tables 3a and 3b).

**TABLE 2b:** Internal consistency and correlation of subscales of Turkish adapted FPSQ‐M.

	FPSQ‐M	Using food to calm	Parent‐led feeding	Persuasive feeding	Feeding on demand
	16 items	5 items	5 items	3 items	3 items
Cronbach's alpha	0.78	0.88	0.81	0.79	0.65
Variance explained (%)	61.42	23.71	17.96	13.00	7.03
Mean ± SD		3.03 ± 1.01	2.95 ± 1.00	1.76 ± 0.86	4.31 ± 0.75
C‐1. Using food to calm		1			
C‐2. Parent‐led feeding		0.048	1		
C‐3. Persuasive feeding		0.282	0.216	1	
C‐4. Feeding on demand		0.346	−0.056	−0.081	1

*Note:* Pearson correlation.

Abbreviations: C, component; FPSQ‐M, Feeding Practices and Structure Questionnaire milking version.

**TABLE 3a:** Rotated component matrix FPSQ‐S.

	C‐1	C‐2	C‐3	C‐4	C‐5	C‐6	KMO	Com	Cronbach's alpha	Cronbach's alpha after CFA^b^
FPSQ‐S Bartlett's test of sphericity							0.843 < 0.01	—	0.87	0.87
REW 5. I encourage my child to eat something by using food as a reward (for example: “If you finish your vegetables, you will get some dessert”)	**0.851**						0.888	0.782	0.89	0.89
REW 6. When my child refuses food they usually eat, I encourage eating by offering a food reward (for example: dessert)	**0.832**						0.890	0.770		
REW 3. I promise my child something other than food if they eat (for example: “If you eat your beans, we can go to the park”)	**0.815**						0.940	0.725		
REW 7. I use desserts as an encouragement to get my child to eat the main course	**0.774**						0.918	0,645		
REW 9. I warn my child that I will take a favorite food away if my child does not eat a food they do not like (for example: “If you don't finish your vegetables, you won't get dessert”)	**0.757**						0.909	0.633		
REW 1. I offer foods to my child as a reward for good behavior	**0.713**		0.390				0.854	0.633		
REW 2. I offer my child their favorite foods in exchange for good behavior	**0.701**		0.363				0.828	0.627		
REW 4. When my child refuses food they usually eat, I encourage eating by offering a non‐food reward (for example: favorite toy or sticker)	**0.548**	0.402					0.947	0.499		
REW 8. I make my child finish the main course before having a dessert	**0.447**	0.313					0.896	0.359		
PERS 6. When my child refuses food they usually eat, I encourage her/him to eat it		**0.813**					0.856	0.693	0.82	0.83
PERS 5. I praise my child after each bit to encourage finishing the food		**0.718**					0.846	0.562		
PERS 7. I play games to make sure my child eats enough		**0.673**					0.873	0.523		
PERS 1. I encourage my child to eat all of the food in front of him/her		**0.664**					0.802	0.497		
PERS 4. I say or do something to show my disapproval of my child for not eating		**0.647**					0.910	0.584		
PERS 2. When my child turns away, I try to get her/him to eat a little bit more		**0.632**					0.875	0.539		
PARENT 1. I carefully control how much my child eats^b^		**0.457**			0.381		0.769	0.440		
FC 4. When my child gets unsettled or is crying, one of the first things I do is give her/him food			**0.759**				0.872	0.608	0.85	0.85
FC 6. I use food to distract my child or keep him/her busy			**0.734**				0.839	0.560		
FC 1. I give my child food to settle him/her even if he/she is not hungry			**0.707**				0.863	0.553		
FC 5. I give my child food to make sure that they do not get unsettled or cry	0.327		**0.701**				0.869	0.603		
FC 2. I offer my child something to eat to make her/him feel better when she/he is unsettled or crying			**0.682**				0.831	0.611		
FC 3. I offer my child something to eat to make her/him feel better when she/he is hurt	0.384		**0.644**				0.883	0.584		
FM 1. My child eats together with other family members.				**0.875**			0.763	0.792	0.83	0.83
FM 3. Whether my child is eating or not, my child sits with the rest of the family when they are having a meal				**0.841**			0.740	0.747		
FM 4. I eat my meals while my child eats				**0.832**			0.735	0.706		
FM 2. My child is given the same foods as the rest of the family (pureed, mashed, chopped)				**0.685**			0.746	0.485		
DEM 4. My child has a set mealtime routine					**0.800**		0.607	0.676	0.66	0.68
DEM 1. My child eats at set times					**0.773**		0.637	0.692		
PARENT 2. I have a rule about how much my child should eat.		0.305			**0.561**		0.741	0.497		
DEM 2. I decide when it is time for my child to eat^b^					**0.539**		0.656	0.337		
PARENT 3. I let my child decide how much she/he eats^a^						**0.731**	0.719	0.612	0.59	0.60
DEM 3. I let my child decide when she/he would like to eat^a^, ^b^						**0.693**	0.489	0.524		
PERS 3. If my child indicates she/he is not hungry I try to get her/him to eat anyway		0.354				**0.513**	0.870	0.519		
PARENT 4. I decide how much my child eats					0.486	**0.495**	0.696	0.553		

*Note:* Bold values indicate the subgroup to which they belong.

Abbreviations: Com, communalities; DEM, feeding on demand (lower score indicates feeding on demand); Extraction Method, Principal Component Analysis; FC, using food to calm; FM, family meal environment; FPSQ‐S, Feeding Practices and Structure Questionnaire‐solid version; PARENT, parent‐led feeding; PERS, persuasive feeding; REW, using (non‐) food rewards; Rotation Method, Varimax with Kaiser normalization.

^a^ Item is reverse coded.

^b^ PARENT 1, DEM 2 and DEM 3 was excluded after CFA.

**TABLE 3b:** Internal consistency and correlation of subscales of Turkish adapted FPSQ‐S.

	FPSQ‐S	Using (non‐) food rewards	Persuasive feeding	Using food to calm	Family meal environment	Feeding on demand	Parent‐led feeding
	31 items	9 items	6 items	6 items	4 items	3 items	3 items
Cronbach's alpha	0.87	0.89	0.83	0.85	0.83	0.68	0.60
Variance explained (%)	59.52	24,44	10,69	8,83	6,12	5,17	4,27
Mean ± SD		1.80 ± 0.76	2.89 ± 0.87	1.59 ± 0.63	3.82 ± 0.84	3.37 ± 0.82	2.01 ± 0.72
C‐1. Using (non‐) food rewards		1					
C‐2. Persuasive feeding		0.435	1				
C‐3. Using food to calm		0.539	0.312	1			
C‐4. Family Meal Environment		0.040	−0.012	−0.021	1		
C‐5. Feeding on demand		0.076	−0.266	0.083	0.222	1	
C‐6. Parent‐led feeding		0.287	0.371	0.276	−0.184	0.243	1

*Note:* Pearson correlation.

Abbreviations: C, component; FPSQ‐S: Feeding Practices and Structure Questionnaire‐solid version.

**TABLE 4:** Test–retest correlations of FPSQ‐M and FPSQ‐S versions.

	Component 1		Component 2		Component 3		Component 4		Component 5		Component 6	
	Retest		Retest		Retest		Retest		Retest		Retest	
	FPSQ‐M	FPSQ‐S	FPSQ‐M	FPSQ‐S	FPSQ‐M	FPSQ‐S	FPSQ‐M	FPSQ‐S	FPSQ‐M	FPSQ‐S	FPSQ‐M	FPSQ‐S
Component 1	0.629**	0.760**										
Component 2			0.456**	0.763**								
Component 3					0.533**	0.846**						
Component 4							0.518**	0.826**				
Component 5									—	0.670**		
Component 6											—	0.453*

*Note:* Pearson correlation.

Abbreviations: FPSQ‐M, Feeding Practices and Structure Questionnaire milking version; FPSQ‐S, Feeding Practices and Structure Questionnaire‐solid version.

*p < 0.05, **p < 0.01.

**TABLE 5a:** Reliability of the Turkish adapted FPSO‐M compared with IOWA.

FPSQ‐M^f^	IOWA‐IFAS			*p* ^d^	Significance^e^
	Positive breastfeeding attitude^a^	Neutral breastfeeding attitude^b^	Positive formula attitude^c^		
C‐1. Using food to calm	3.20 (2.10)	3.00 (1.60)	1.90 (1.75)	0.12	NA
C‐2. Parent‐led feeding	3.00 (1.58)	3.16 (1.17)	2.83 (2.08)	0.39	NA
C‐3. Persuasive feeding	1.33 (1.00)	1.66 (1.33)	1.00 (0)	0.02	c‐a: 0.05 c‐b: 0.01 a–b: 0.20
C‐4. Feeding on demand	4.50 (1.00)	4.50 (0.81)	4.25 (2.06)	0.241	NA

Abbreviations: C, component; FPSQ‐M, Feeding Practices and Structure Questionnaire milking version; IOWA‐IFAS, IOWA Infant Feeding Attitude Scale.

^c‐a^ Compared to a positive attitude toward breastfeeding, parents who used persuasive feeding methods had a significantly lower positive attitude toward formula.

^d^ Kruskal–Wallis test.

^e^ Bonferroni correction.

^f^ Median‐IQR.

**TABLE 5b:** Reliability of the Turkish adapted FPSO‐S compared with PFSQ.

FPSQ‐S	PFSQ				
	Emotional feeding SS	Instrumental feeding SS	Encouragement SS	Strict controlled feeding SS	Tolerant controlled feeding SS
C‐1. Using (non‐) food rewards	0.610**	0.738**	0.254**	0.048	0.138*
C‐2. Persuasive feeding	0.280**	0.294**	0.546**	0.289**	0.079
C‐3. Using food to calm	0.669**	0.500**	0.131*	−0.018	0.172**
C‐4. Family meal environment	−0.083	−0.132*	0.243**	0.323**	0.151*
C‐5. Feeding on demand	−0.010	0.036	0.183**	0.309**	−0.113
C‐6. Parent‐led feeding	0.169**	0.271**	−0.064	0.021	−0.286**

*Note:* Pearson correlation.

Abbreviations: C, component; FPSQ‐S, Feeding Practices and Structure Questionnaire‐solid version; PFSQ, Parental Feeding Style Questionnaire; SS, subscale.

*p < 0.05, **p < 0.01.

### 3.3 Confirmatory Factor Analysis (CFA)

**FIGURE 1:**
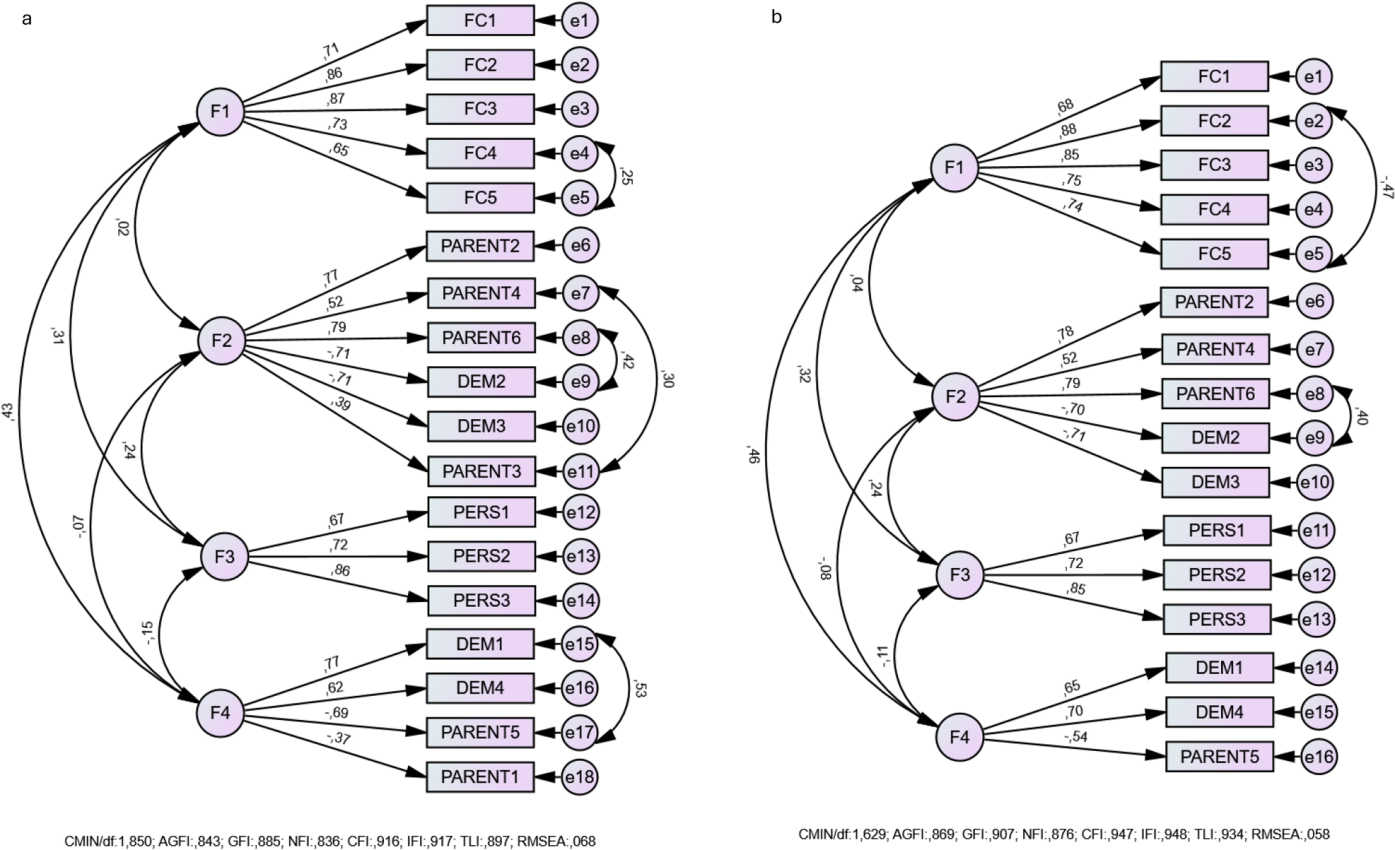
Confirmatory factor analysis of FPSQ‐M original (a) vs. Turkish versions (b).

**FIGURE 2:**
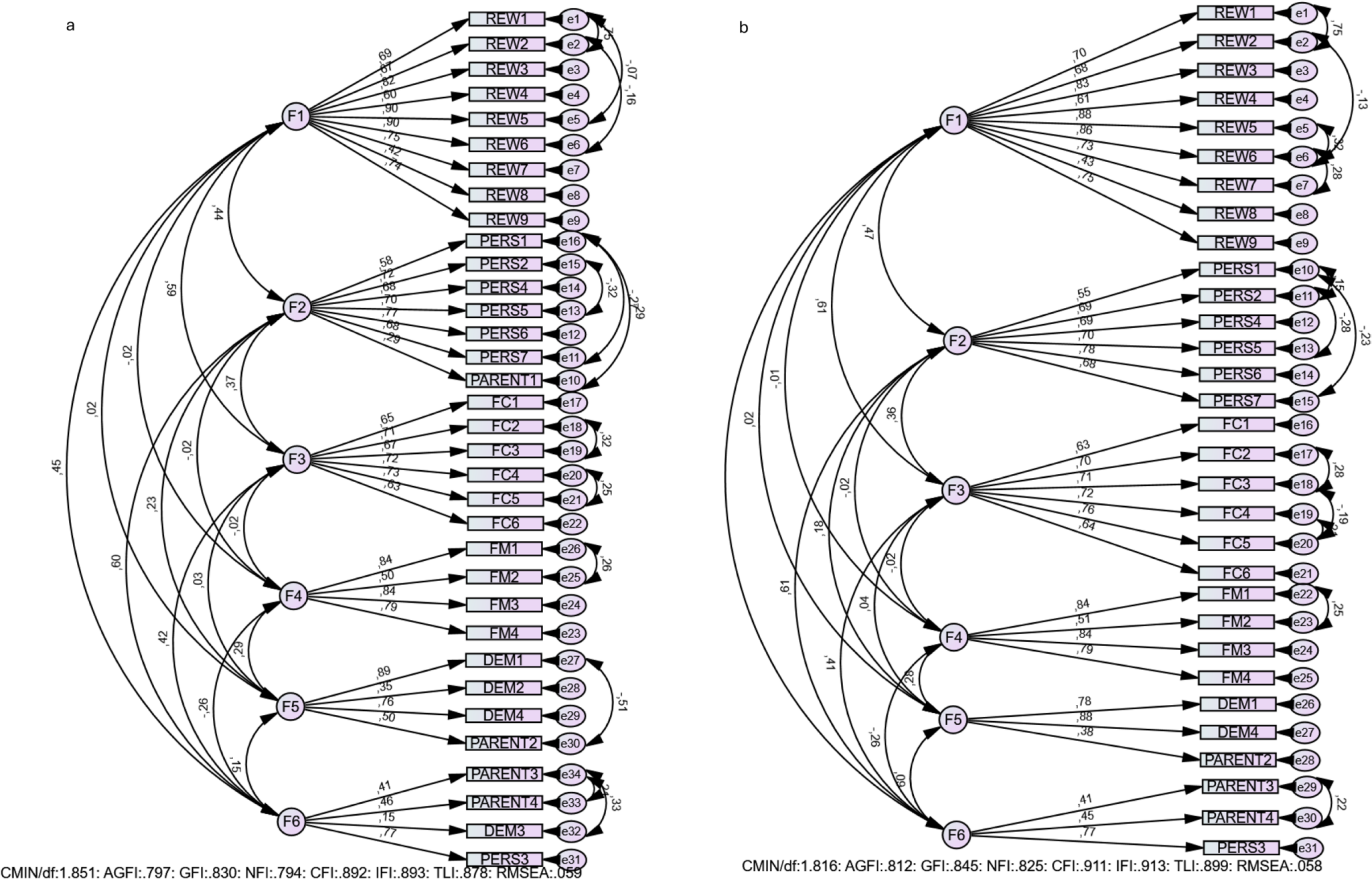
Confirmatory factor analysis of FPSQ‐S original (a) vs. Turkish versions (b).

The 2 items from FPSQ‐M (PARENT 1 and PARENT 3) and 3 items from FPSQ‐S (PARENT 1 and DEM 2 and DEM 3) had factor loadings close to or below 0.40 as in the original article (Jansen et al. 2021). These items were excluded from CFA. The final version of the questionnaires consists of 16 items in FPSQ‐M and 31 items in FPSQ‐S version (Figure 1, 2).

#### 3.3.1 Confirmatory Factor Analysis of FPSQ‐M Version

The fit indices for the FSPQ‐M demonstrated an acceptable model fit. The chi‐square value normalized by degrees of freedom (CMIN/df) was 1.829, indicating a good fit (values < 3 are considered acceptable) (Schumacker and Lomax 2004). The root mean square error of approximation (RMSEA) was 0.058, which is within the range of a good model fit (< 0.06) (Bentler 1990). The comparative fit index (CFI) and incremental fit index (IFI) were 0.947 and 0.948, respectively, both exceeding the recommended threshold of 0.90 (Bentler 1990). Similarly, the Tucker–Lewis index (TLI) was 0.934, further supporting the adequacy of the model (Hu and Bentler 1999). The goodness‐of‐fit index (GFI = 0.907), adjusted goodness‐of‐fit index (AGFI = 0.859), and normed fit index (NFI = 0.876) were all above acceptable or near acceptable levels (Tabachnick et al. 2013). These results support the structural validity of the Turkish version of the FSPQ‐M (Figure 1).

#### 3.3.2 Confirmatory Factor Analysis of FPSQ‐S Version

The fit indices for the Turkish version of the FSPQ‐S demonstrated an acceptable model fit. The chi‐square value normalized by degrees of freedom (CMIN/df) was 1.816, indicating a good fit (Schumacker and Lomax 2004). The root mean square error of approximation (RMSEA) was 0.058, which is within the range of a good model fit (< 0.06) (Bentler 1990). The comparative fit index (CFI) and incremental fit index (IFI) were 0.911 and 0.913, respectively, both exceeding the recommended threshold of 0.90 (Bentler 1990). Similarly, the Tucker–Lewis index (TLI) was 0.899, closely approaching the acceptable cutoff of 0.90 (Hu and Bentler 1999). However, the adjusted goodness‐of‐fit index (AGFI = 0.812), goodness‐of‐fit index (GFI = 0.845), and normed fit index (NFI = 0.825) were slightly below the desired threshold of 0.90, which may indicate minor deviations from a perfect fit (Tabachnick et al. 2013). Despite this, the overall fit indices support the structural validity of the Turkish version of the FSPQ‐S (Figure 2).

### 3.4 Test–Retest

Three weeks after the initial application, both questionnaires were re‐administered to a subset of 30 participants who had completed the initial forms. The test–retest reliability of both versions of the FPSQ was assessed using Pearson correlation coefficients for the components. The results for both versions demonstrated acceptable stability across time, with all components showing statistically significant and positive correlations (the test–retest Pearson correlation coefficients for the components of the FPSQ‐M version were 0.629, 0.426, 0.533 and 0.518; and for the components of the FPSQ‐S version were 0.760, 0.763, 0.846, 0.826, 0.670, and 0.453, respectively). These findings support the external reliability of both the FPSQ‐M and FPSQ‐S for assessing feeding practices in Turkish children, with all components demonstrating significant and moderate to strong test–retest reliability across the sample (Table 4).

### 3.5 Reliability

**FIGURE 3:**
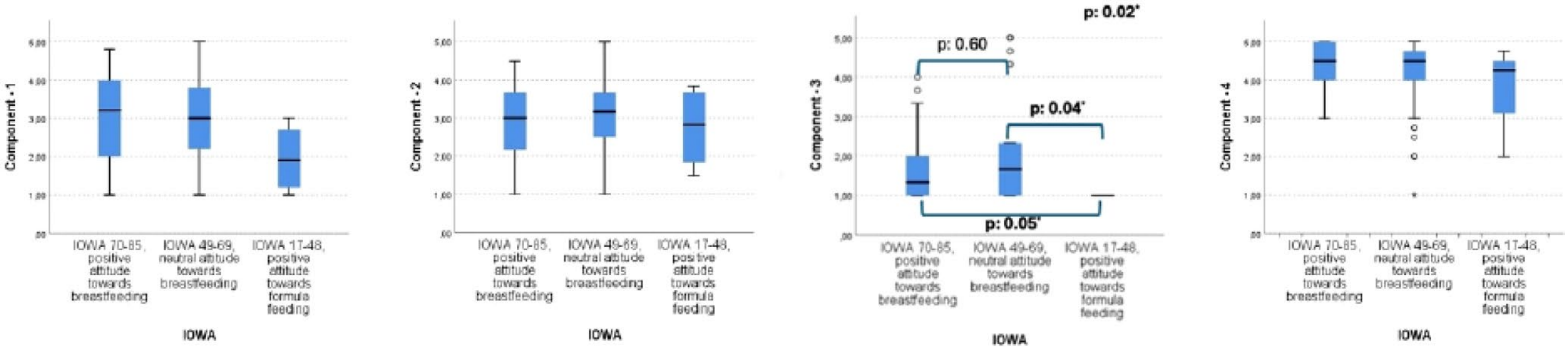
Reliability of the FPSO‐M compared with IOWA.

When comparing the relationship between components of FPSQ‐M with the Turkish adapted, reliable, and validated IOWA‐IFAS questionnaire's subscale, it was found that positive formula attitude of parents was significantly lower in parents who are following persuasive feeding methods (Table 5a, Figure 3).

When the subscales of the FPSQ‐S were compared with the PFSQ, which has been subjected to reliability and validity testing in our country and used for the same age group, significant positive high correlations were found in the subscales containing similar items. The correlations between the subscales of FPSQ‐M's “Using food rewards” and “Using food to calm” with PFSQ's “Emotional feeding” and “Instrumental feeding” and FPSQ‐M's “Persuasive feeding” with PFSQ's “Encouragement” was positively significant (*r* > 0.5, *p* < 0.01 for all), with an effect size of Cohen's *r* ^2^ > 0.25, indicating that more than 25% of the variance in PFSQ's related subscale can be explained by the FPSQ's related subscales (Table 5b).

## 4 Discussion

To measure families' feeding practices in terms of predominantly breastfeeding (FPSQ‐M) and (Semi‐)Solid Foods (FPSQ‐S) in early infancy, the subscales were developed using the original FPSQ (Jansen et al. 2014, 2021). The results of this cultural adaptation study suggest a four‐factor solution for the FPSQ‐M version and a six‐factor solution for the FPSQ‐S in a sample of Turkish parents with infants aged 0–24 months. The final Turkish version of the FPSQ‐M is composed of 16 items (alpha of 0.78) with acceptable reliability, and FPSQ‐S is composed of 36 items (alpha of 0.87) with good reliability. Factor structure and items included in the factors differed from the original study, highlighting the cultural factors related to parental perceptions about nutrition.

The results of the four‐component FPSQ‐M version are consistent with psychometric guidelines, which consider Cronbach's alpha values above 0.70 to indicate acceptable reliability (Bartlett 1950; Mallery and George 2000). In addition, the factorial structure supports the multidimensional nature of feeding practices in line with previous research, emphasizing the importance of parent–child interaction and context in feeding behaviors (Jansen et al. 2012, 2021; Mitchell et al. 2013). Two questions were excluded in the Turkish version (PARENT 3. I carefully control how much my baby feeds, PARENT 1. When deciding how much to feed my baby, I rely on how hungry he is). After excluding these two questions, the RMSEA, CFI, and TLI measures showed that the model had an acceptable fit to the data, similar to the original FPSQ‐M version (Jansen et al. 2021). These two items cannot be identified by parents as belonging to the parent‐controlled feeding or the Feeding on demand subscale. In Türkiye, mothers are traditionally seen as the primary caregivers, especially during infancy (Akçınar 2017). Early initiation and exclusive breastfeeding are highly valued and widely practised as cultural practices by the family, close social network, and religious community (Demirtas et al. 2012; Hacettepe Üniversitesi Nüfus Etütleri Enstitüsü 2021; Eskici and Karahan Yilmaz 2022). Breastfeeding is often used as soothing response to infant crying, which is interpreted as a sign of hunger. As a result mothers may be more sensitive to hunger cues than to satiety cues, possibly because they may blame themselves when they are unable to soothe their infant (Muller et al. 2023). This may be the reason for the nondiscriminatory nature of these two items. Although the distribution of items across factors was found to be the same for the “Persuasive feeding” and “Using food to calm” subscales, the items on the “Parent‐led feeding” and “Feeding on demand” subscales were redistributed in the Turkish version. The internal consistency of the subscales of the Turkish version of the FPSQ‐M was found to be higher than that of the original version (Jansen et al. 2021). As noted in the original article, there is more complexity in capturing the actual feeding interaction that occurs in infancy (Jansen et al. 2021) although both subscales had acceptable internal consistency. It was not surprising that the internal consistency of the feeding on demand subscale was low, as Turkish parents are culturally more likely to control the structure of feeding while following hunger cues, especially in early infancy. As noted in the original article, the benefits of feeding on demand—where the infant determines the timing of feedings without a fixed routine—remain unclear, particularly in early developmental stages (Jansen et al. 2021). In addition, responsive feeding not only involves the mother's willingness to feed her baby in response to the baby's cues but also emphasizes that feeding sessions are an important opportunity to nurture the bond of love, comfort, and trust between mother and baby (UNICEF UK 2013). For successful breastfeeding, it is recommended to follow the baby's cues and latch on to the breast whenever the baby indicates a desire to nurse. This allows the baby to regulate the milk supply, which can increase the amount of breast milk. In this bidirectional relationship, crying and fussing are observed as cues from the baby, and a routine of responding to each cry is established. During the first 6 months of life, when mothers are named as the primary caregivers in our country, the inability to satisfy their baby's hunger or crying can have a significant emotional impact, with mothers often experiencing feelings of frustration, failure, inadequacy, and self‐blame (Muller et al. 2023). As a result, these vulnerable caregivers may be more likely to adopt feeding practices that focus on the structure of feeding and may be particularly sensitive to the timing of hunger cries in their efforts to soothe their babies. Despite our study groups' higher educational attainment compared to the general population, it was interesting that parents did not discriminate the reverse‐coded items on both versions of the FPSQ. Although the reverse‐coded items were re‐coded at the analysis stage, they had a negative factor load. In line with this, we decided to code them as normal in the context of the full fit with the remaining items of the subscales (DEM 3. I decide when it is time for my baby to have a feed, DEM 2. I feed my baby at set times, PARENT 5. I let my baby decide how much he feeds). These findings indicate that even from an early age, these factors may cluster together, representing a group of (non‐) responsive feeding practices. Although external reliability was not assessed in the original article (Jansen et al. 2021), our test–retest results for the FPSQ‐M version demonstrate acceptable stability over time. These findings support the external reliability of the FPSQ‐M in assessing feeding practices among Turkish children, with all components showing moderate to strong test–retest reliability. This suggests that the FPSQ‐M is a stable and reliable tool for evaluating feeding practices in the Turkish population.

The results of the six‐component FPSQ‐S version are consistent with psychometric guidelines, which consider Cronbach's alpha values above 0.80 to indicate good reliability (Bartlett 1950; Mallery and George 2000). The distribution of items across factors was found to be the same for the “Using (non‐) food rewards” “Family Meal Environment” and “Using food to calm” and except for one item “Persuasive feeding” subscales. However, the items on the “Parent‐led feeding” and “Feeding on demand” subscales were redistributed in the Turkish version as in the milk feeding version. Although the internal consistency of the feeding on demand and Parent‐led Feeding subscales was lower compared to the original version, it remained acceptable. In addition, the internal consistency of the other subscales in the Turkish version of the FPSQ‐S was similar to that of the original version (Jansen et al. 2021). In the Turkish version of the FPSQ‐S, three items were excluded (PARENT 1. I carefully control how much my child eats, DEM 2. I decide when it is time for my child to eat; and DEM 3. I let my child decide when she/he would like to eat). After excluding these three items, the RMSEA, CFI, and TLI measures showed that the model had an acceptable fit to the data, similar to the original FPSQ‐S version (Jansen et al. 2021). Our test–retest results for the FPSQ‐S version demonstrate significant and strong stability over time. The observed significant correlations between the subscales of the FPSQ‐S and PFSQ supported the convergent validity of the FPSQ‐S in Turkish context (Özçetin et al. 2010; Wardle et al. 2002). In particular, the alignment of subscales such as “Using food rewards” and “Using food to calm” with “Emotional feeding” and “Instrumental feeding” which reflects similar practices of using food to manage emotions; and “Persuasive feeding” with “Prompting/encouragement” indicating a common focus on encouraging or influencing children's food intake through parental prompting or persuasion, highlights overlapping constructs between these instruments. These findings contribute to the literature by validating a comprehensive measure to assess feeding practices in Türkiye and providing a novel cross‐validation approach between two widely used instruments (Özçetin et al. 2010; Wardle et al. 2002). This adds depth to the understanding of culturally relevant feeding practices in early childhood. The results further suggests that the FPSQ‐S is a stable and reliable tool for evaluating feeding practices among Turkish parents predominantly feeding with (semi‐) solids.

The limitations of the study can be grouped into two main themes. As this is the first and only study to adapt both versions of the FPSQ into another language, there is limited data on other cultural validation studies with which to compare our data. Also, we did not collect data to assess feeding difficulties or families' perceptions of their children's feeding as a problem, so we were not able to compare our data in this regard.

The Turkish language adapted FPSQ‐M with a 16‐item distribution, a four‐factor structure, and the FPSQ‐S with a 31‐item distribution, a six‐factor structure, are reliable questionnaires for measuring feeding practices among Turkish children aged between 0 and 24 months. Future studies are needed to assess the impact of cultural feeding practices in early infancy on feeding problems. In addition, the use of these culturally adapted questionnaires will provide an opportunity to advise parents to follow responsive feeding practices throughout infancy and childhood, whereas referring to more culturally sensitive feeding practices.

## Author Contributions

**Mahmut Caner Us:** conceptualization (lead), data curation (lead), investigation (lead), methodology (lead), software (lead), writing – original draft (lead), writing – review and editing (equal). **Hatice Ezgi Baris:** conceptualization (equal), data curation (equal), formal analysis (equal), methodology (equal), resources (equal), validation (equal), writing – review and editing (lead). **Elif Öztürk:** conceptualization (equal), data curation (equal), formal analysis (equal), methodology (equal), supervision (equal), validation (equal), writing – review and editing (equal). **Ayşe Şahin:** data curation (equal), investigation (equal), methodology (equal), supervision (equal), validation (equal), writing – review and editing (equal). **Gökçe Nizam:** data curation (equal), investigation (equal), visualization (equal), writing – review and editing (equal). **Betül Şenyürek:** investigation (equal), validation (equal), visualization (equal), writing – review and editing (equal). **Perran Boran:** conceptualization (equal), investigation (equal), methodology (equal), supervision (lead), validation (equal), visualization (equal), writing – review and editing (equal).

## Conflicts of Interest

The authors declare no conflicts of interest.

## Data Availability

The data that support the findings of this study are available on request from the corresponding author.
